# Seasonality of newly notified pulmonary tuberculosis in Japan, 2007–2015

**DOI:** 10.1186/s12879-019-3957-8

**Published:** 2019-06-06

**Authors:** Toshie Manabe, Jin Takasaki, Koichiro Kudo

**Affiliations:** 1 Division of Community and Family Medicine, Center for Community Medicine, Jichi Medical University, 333-1 Yakushiji, Shimotsuke, Tochigi Japan; 2 Waseda University Organization of Regional and Inter-Regional Studies, Tokyo, Japan; 3 Department of Hygiene and Public Health, Teikyo University School of Medicine, Tokyo, Japan; 4 Department of Respiratory Medicine, National Center for Global Health and Medicine, Tokyo, Japan

**Keywords:** Tuberculosis, Seasonal variation, Epidemic peak, Latent tuberculosis, Roger’s test

## Abstract

**Background:**

The seasonality of pulmonary tuberculosis (TB) incidence may indicate season-specific risk factors that could be controlled if they were better understood. The aims of this study were to elucidate how the incidence of TB changes seasonally and to determine the factors influencing TB incidence, to reduce the TB burden in Japan.

**Methods:**

We assessed the seasonality of newly notified TB cases in Japan using national surveillance data collected between 2007 and 2015. To investigate age and sex differences, seasonal variation was analyzed according to sex for all cases and then by stratified age groups (0–4, 5–14, 15–24, 25–44, 45–64, 65–74, and ≥ 75 years). We used Roger’s test to analyze the cyclic monthly trends in seasonal variation of TB incidence.

**Results:**

A total of 199,856 newly notified TB cases (male, 62.2%) were reported over the past 9-year period. Among them, 60.6% involved patients aged ≥65 years. Overall, the peak months of TB incidence occurred from April to October, excluding September. In the analysis stratified by age group, a significant seasonal variation in TB cases was observed for age groups ≥15 years, whereas no seasonal variation was observed for age groups ≤14 years. For female patients aged ≥25 years, the peak TB epidemic period was seen from June to December, excluding November. Male patients in the same age groups exhibited declining TB incidence from September to March.

**Conclusions:**

TB incidence exhibits seasonality in Japan for people aged > 15 years and peaks in summer to fall. Monthly trends differ according to age and sex. For age groups ≥25 years, cases in women showed longer peaks from June to December whereas cases in men declined from September to December. These results suggest that the seasonality of TB incidence in Japan might be influenced by health checkups in young adults, reactivation of latent TB infection with aging, and lifestyle habits in older adults. These findings can contribute to establishing the potential determinants of TB seasonality in Japan.

## Background

Tuberculosis (TB) continues to present a major global health burden. The World Health Organization has estimated that there were 6.1 million new TB cases reported and 1.8 million TB deaths globally in 2016 [1]. In Japan, the incidence rate of newly notified TB cases in 2016 was 13.9 per 100,000 population [2, 3], which is categorized as an intermediate TB-burden country. In contrast, the most of industrialized countries are categorized as low-burden TB countries [1–3]. A better understanding of whether Japan has specific reasons for remaining the intermediate burden of TB is urgently required, to reduce the number of TB cases in the country.

The seasonality of TB incidence has been widely reported in different parts of the world, and understanding season-specific risk factors for active TB can help in developing national TB control policies; however, previous studies have reported conflicting incidence peaks of TB in spring, summer, and winter [4–8]. Although the exact mechanisms underlying the fluctuation of TB during particular times of the year remain unclear, several studies have suggested that environmental factors (including cold weather, sunlight, air pollution) and social factors (such as crowding, person-to-person contact, and health care-seeking behavior) contribute to the seasonality of TB incidence [4–8]. However, TB occurs not only via a recent infection that progresses rapidly but also via reactivation of TB infection later in life, after prolonged latency [8]. In Japan, a super-aging society, the frequency of TB cases among patients aged ≥80 years was 37.7% of all newly registered TB cases to the Japanese TB patient registration system in 2016, and the number of latent TB cases has been increasing in older populations [2, 3]. Although a possible link between vitamin D deficiency and latent TB has been reported [9–11], active TB can arise as a consequence of reactivation of latent TB infection following compromise of the anti-mycobacterial immune response. Reactivation of latent TB infection in older adults may be influenced by decreased immunity caused by aging. However, season-specific risk factors for newly notified TB cases in Japan, including reactivation of latent TB infection, have not yet been fully investigated. In addition, a previous report on TB seasonality in Japan indicated that the trend of seasonality varies according to the form of TB, including childhood TB (age 0–14 years), pleural TB in younger populations, smear-positive TB, and lymph node TB [12]. However, previous studies have not included statistical analyses to test the cyclic trend of TB seasonality.

The aims of the present study were to assess whether seasonality of new TB cases exists in Japan and to characterize the patterns of seasonality in newly notified TB cases. The results may assist with determining the seasonal factors related to new TB cases in Japan and could help to explain why Japan remains the intermediate-burden TB country.

## Methods

We used data obtained from TB surveillance by the Ministry of Health, Labour, and Welfare in Japan and the Tuberculosis Surveillance Center, between 2007 and 2015 [13, 14]. In Japan, TB has been classified as a Category 2 Infectious Disease according to the “Act on the Prevention of Infectious Diseases and Medical Care for Patients with Infectious Diseases” (the Infectious Diseases Control Law), enforced 2007 [15]. Under this regulation, if physicians diagnose a patient with tuberculosis, they are required to immediately notify the public health center. This obligation applies for all TB patients who visit any hospital in Japan, regardless of nationality or ethnicity. Along with this law, a new computerized tuberculosis surveillance system was launched in 2007, which contains a more detailed and more accurate TB registration system than the previous system, including rapid registration and unduplicated registration. Therefore, 100% of patients with TB who visited hospitals in Japan between 2007 and 2015 could be included in this study. Under this condition, in this study, the newly notified TB cases was defined as a newly registered TB case in Japanese TB patient registration system.

We used data on newly notified TB cases in Japan, which are defined according to a combination of the following: clinical presentations and respiratory symptoms for TB); detection of *M. tuberculosis* complex; and chest radiographs, including computed tomography, based on the Guideline of Clinical Management of Tuberculosis [16].

We divided the year into four seasons of 3 months each: spring (March–May), summer (June–August), fall (September–November), and winter (December–February), using definitions set by the Japan Meteorological Agency [17].

To observe age and sex differences, TB seasonality was evaluated according to sex for all cases and then by stratified age groups: 0–4, 5–14, 15–24, 25–44, 45–64, 65–74, and ≥ 75 years.

Ethical approval and the consent by the participants was waived by the ethics committee of Teikyo University for this study, which used open access, anonymized statistical and epidemiological information.

### Statistical analyses

To calculate seasonal variations in the incidence data between 2007 and 2015, we summed the number of newly notified TB cases in each month for each year. We used the significance test for seasonal variation proposed by Roger, which evaluates the significance of cyclic trends according to the efficient score vector calculated for one seasonal peak [18]. This statistical method determines a simple harmonic cyclic trend by dividing a circle into 12 equal sectors and plotting the monthly frequencies as coordinates in the corresponding sectors of the circle [18–20].

Data analyses were conducted using SAS version 9.4 (SAS Institute Inc., Cary, NC, USA). All *p* values were two-tailed, and *p* < 0.05 was considered significant.

## Results

### Characteristic seasonal distributions of TB cases in Japan

**Fig. 1:**
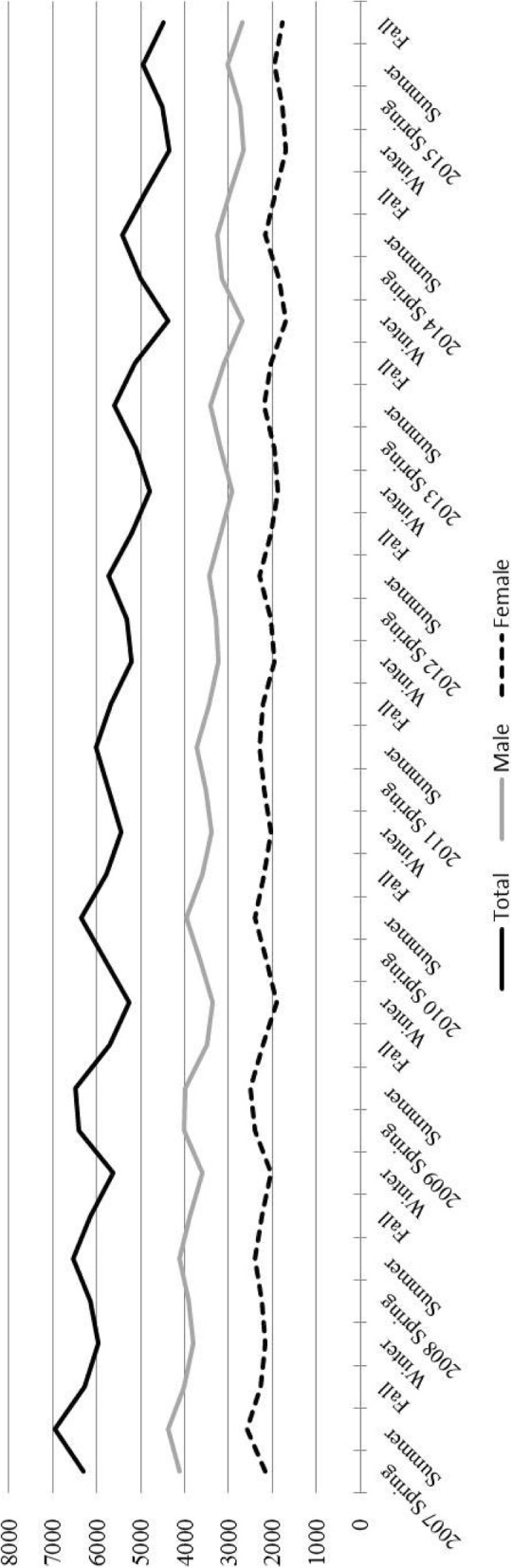
Distribution of the number of newly notified pulmonary tuberculosis cases in Japan, 1998–2015. Black, total cases; dark gray, male cases; light gray, female cases

From 2007 and 2015, a total of 199,856 newly notified pulmonary TB cases (male patients, 62.2%) were reported in Japan that were eligible for the present study. The distributions of the number of newly notified pulmonary TB cases in Japan between 2007 and 2015 during each season, according to sex, are shown in Fig. 1. The number of TB cases tended to decrease annually and the shapes of epidemic waves were similar in both sexes, presenting a high in summer and a low in winter seasons (Fig. 1). Table 1 shows the frequency of TB cases in each season according to sex and age group. The highest frequency of TB cases in each season was observed in the age group ≥75 years (Table 1). Although the epidemic peak among all cases appears high in summer and low in winter (Fig. 1), the peaks vary when presented according to stratified age groups: spring in the age group 15–24 years, summer in the group aged 25–44 years, winter for ages 45–64 years, fall and winter in the age group 65–74 years, and fall in the group aged ≥75 years. The frequency of TB cases in the age groups ≤4 years and 5–14 years were low and nearly the same in each season.

**Table 1:** Frequency of newly notified tuberculosis cases in Japan, 2007–2015

	All	Spring	Summer	Fall	Winter
*n* (%)	*N* = 199,856	50,203 (25.1)	54,181 (27.1)	49,307 (24.7)	46,165 (23.1)
Gender					
Male	124,228 (62.2)	31,512 (62.8)	33,386 (61.6)	30,359 (61.6)	28,971 (62.8)
Female	75,628 (37.8)	20,795 (38.4)	20,795 (38.4)	18,948 (38.4)	17,194 (37.2)
Age					
≤ 4 y	302 (0.2)	81 (0.2)	78 (0.1)	74 (0.2)	69 (0.1)
5–14 y	374 (0.2)	90 (0.2)	113 (0.2)	89 (0.2)	82 (0.2)
15–24 y	7393 (3.7)	2122 (4.2)	2163 (4.0)	1660 (3.4)	1448 (3.1)
25–44 y	30,714 (15.4)	7633 (15.2)	8618 (15.9)	7448 (15.1)	7015 (15.2)
45–64 y	39,922 (20.0)	10.083 (20.1)	10,893 (20.1)	9595 (19.5)	9351 (20.3)
65–74 y	33,467 (16.8)	8420 (16.8)	8833 (16.3)	8370 (17.0)	7844 (17.0)
≥ 75 y	87,310 (43.8)	21,254 (42.3)	23,542 (43.5)	22,182 (45.0)	20.332 (44.0)

Month of each season was defined as spring (March–May), summer (June–August), fall (September–November), and winter (December–February) according to the definitions by the Japan Meteorological Agency [16]

### Seasonality of TB cases in Japan according to age and sex

**Fig. 2:**
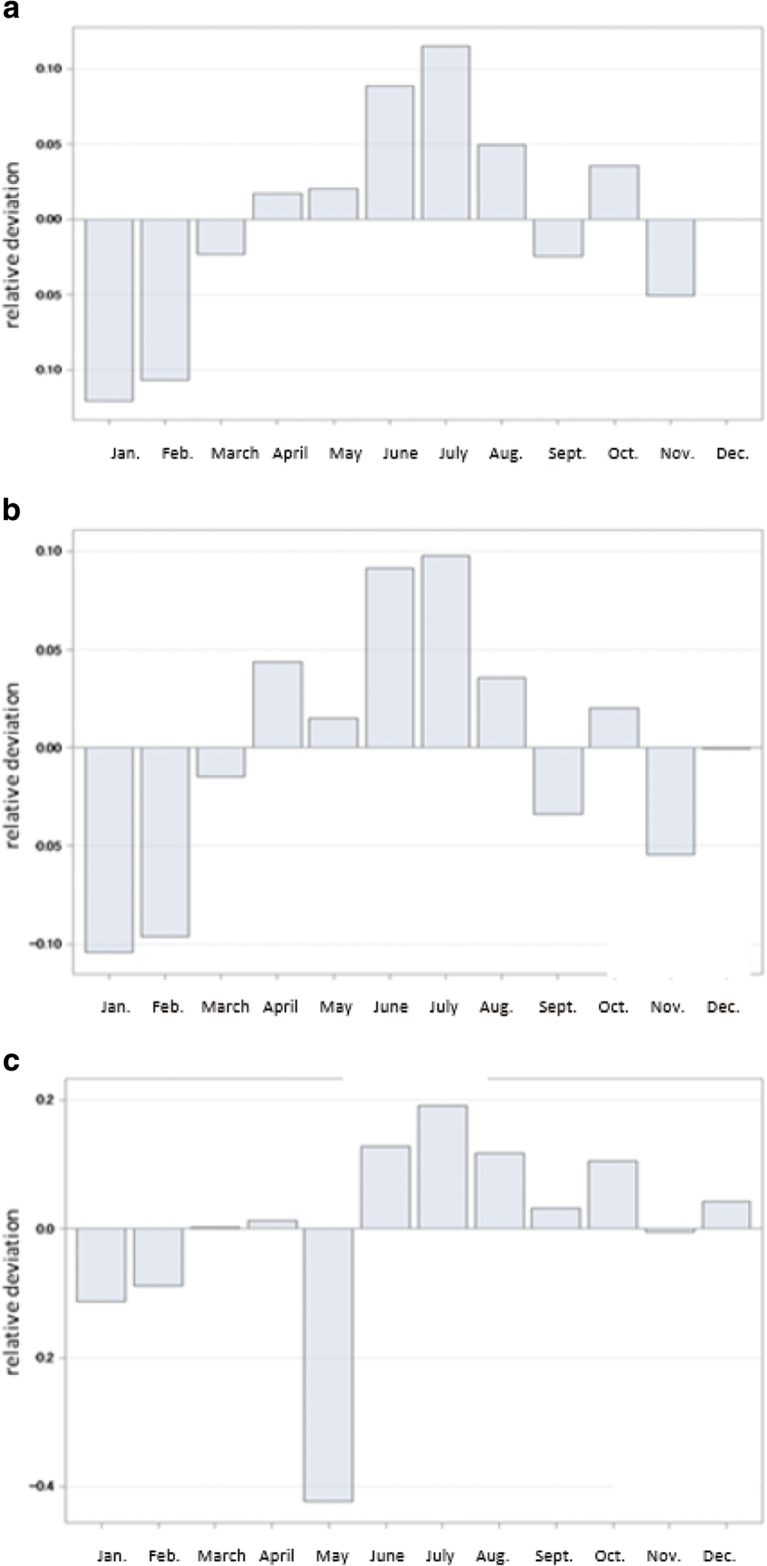
Seasonal variation among tuberculosis cases in Japan, 2007–2015. (**a**) Total cases; (**b**) male cases; (**c**) female cases

Seasonality in newly notified TB cases in Japan among all cases and according to sex is shown in Fig. 2. The significant seasonal variations were evaluated in all cases and in male and female groups, using Roger’s test (*p* < 0.001). Overall, the peak months were April–October in all groups (Fig. 2a). Although similar epidemic peaks were observed in male and female groups, the monthly peak started earlier in the male group (Fig. 2b) than in the female group (Fig. 2c). The highest peak month was July in the female group and June–July in the male group.

**Fig. 3:**
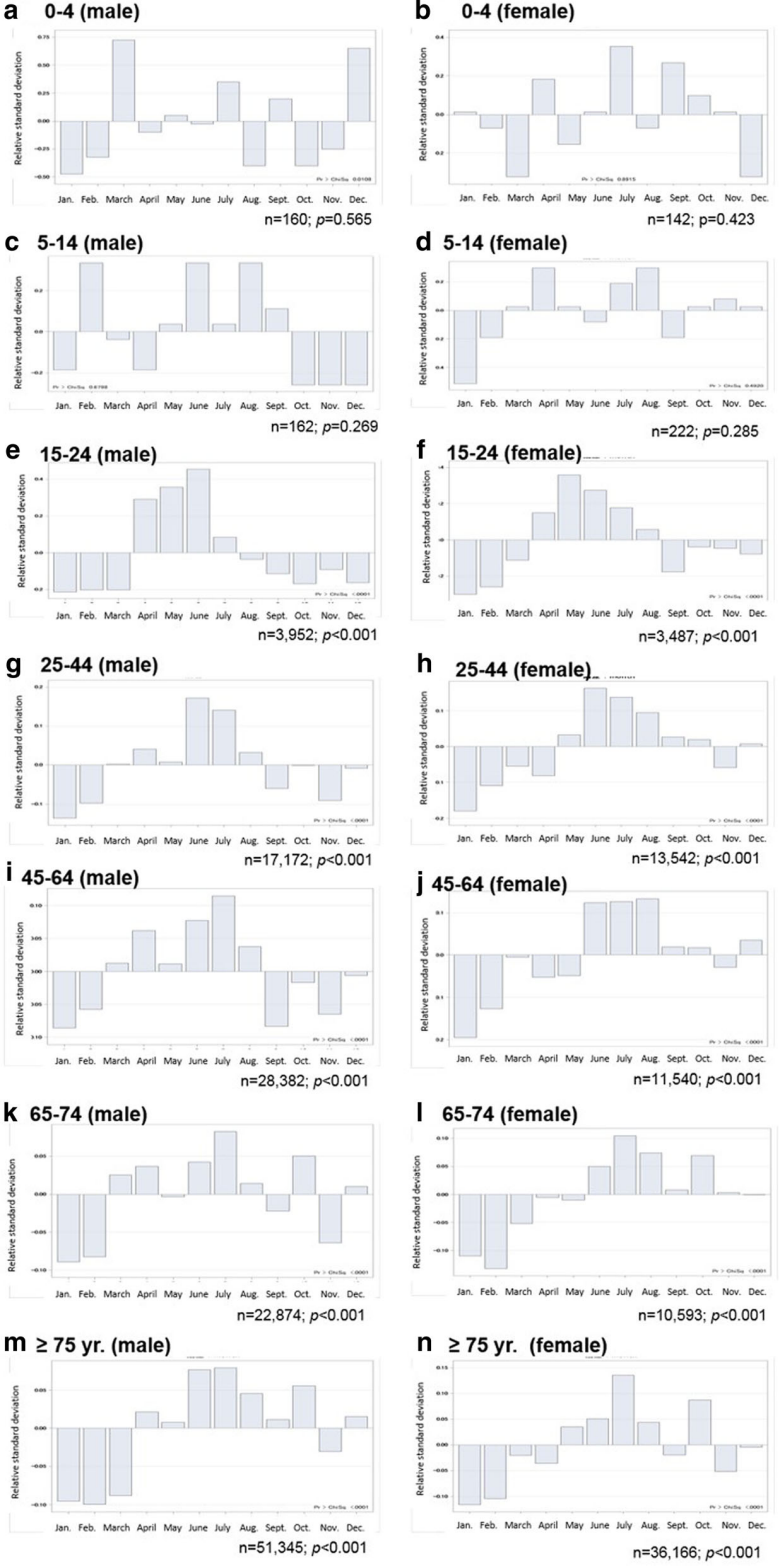
Seasonal variation among tuberculosis cases in Japan according to age group and sex, 2007–2015. (**a**) male, age 0-4 yr.; (**b**) female, age 0-4 yr.; (**c**) male, age 5-14 yr.; (**d**) female, age 5-14 yr.; (**e**) male, age 15-24 yr.; (**f**) female, age 15-24 yr.; (**g**) male, age 25-44 yr.; (**h**) female, age 25-44 yr.; (**i**) male age 45-64 yr.; (**j**) female, age, 45-64 yr.; (**k**) male, age 65-74 yr.; (**l**) female, age 65-74 yr.; (**m**) male, age ≥75 yr.; (**n**) female, age ≥75 yr

Figure 3 shows the evaluation of newly notified TB cases in stratified age groups, according to sex. TB cases among age groups ≥15 years exhibited significant seasonal variation (Fig. 3e–3n) whereas the age groups 0–4 and 5–14 years showed no significant seasonal variation in either male (Fig. 3a, c) or female groups (Fig. 3b, d). The highest peak incidence of TB cases in age groups ≥15 years differed according to age and sex. TB cases among females aged 25–44 years started to increase in May and the epidemic period continued through December, with the exception of November (Fig. 3h). This tendency was similar in higher age groups among females (Fig. 3j, l, n). On the contrary, among males aged 25–44 years, the lowest peak month started in September, and incidence remained low until February (Fig. 3h). This trend was seen in male age groups 45–64 years and 65–74 years, except in October for males aged 65–74 years. The monthly trend in males aged ≥75 years (Fig. 3m) was similar to that in females in the same age group; however, the lowest peak months among males were observed in January–March whereas those among females were only in January and February (Fig. 3n).

## Discussion

The present study revealed seasonality of newly notified TB cases in Japan, particularly during the late spring and summer seasons. However, TB cases in patients aged ≤15 years, who may have been infected recently, demonstrated no significant seasonality. Moreover, monthly trends differed according to age group and sex. The highest peaks of monthly incidence in patients aged ≥15 years were concentrated in June and July. The epidemic period tended to be longer with increasing age, indicating that patients may have experienced reactivation of latent TB infection.

Studies in many counties have evaluated the seasonality of newly notified TB cases [4–12, 21–30]. These reports have indicated various factors that can explain the mechanisms underlying TB seasonality, including patient lifestyle, temperature, climate region, sunlight, vitamin D deficiency, and air pollution [4–12, 21–30]. In Japan, although TB incidence has gradually declined since 2007 (Fig. 1), the seasonal variation of TB incidence was similar over the observed 9 year period (Fig. 1. 2). The overall age distribution of newly notified TB cases has become concentrated in older adults. Among newly notified TB cases in 2007–2015, 60.6 and 43.8% of all cases were in patients aged ≥65 and ≥ 75 years, respectively (Table 1). A plausible reason for the large number of TB cases seen in older populations is that Japan presently has the oldest population in the world [31]. This high number of older people relates to an increase in the population who experienced latent TB infection in previous life years. Historically, Japan had been considered a high-burden TB country, and TB was the number one cause of death approximately 50 years ago [32]. It is thought that most TB cases in older populations may be caused by reactivation of latent TB infection contracted at a young age. Thus, the seasonality of all TB cases may be related to the seasonality for reactivation of latent TB infection. Previously, a study in Japan indicated that the seasonality of TB notification differed among various forms of TB, including pleural TB, smear-positive pulmonary TB, and lymph node TB [12]. Although reactivation of latent TB may present some of these forms, the seasonality of reactivated latent TB differs from the types of TB in the previous report. In addition, previous studies have only investigated the number of TB cases according to the different forms of TB and have not statistically examined seasonal variation using Roger’s test.

Therefore, in this study, we assessed the seasonal variation of newly notified TB cases in Japan according to stratified age groups, using the Roger’s test. We found no significant seasonality in TB cases among age groups < 5 and 5–14 years; however, significant seasonality was seen in the remaining age groups (Fig. 3). Younger people generally acquire TB as a new, recent infection that progresses over a short period of time to onset. Therefore, the delay between disease onset and diagnosis can be short in young patients [33]. In addition, the chances of contracting TB infection are more limited in children than adults. A study in Japan indicated that 60% of infants with TB were infected from within their families [34]. These results indicate that the incidence of pediatric TB may be more strongly influenced by host-specific factors rather than season-specific risk factors. Currently, the burden of pediatric TB cases is low in Japan (Table 1); however, one study indicated that the frequency of mild pediatric TB cases has been increasing [35]. Therefore, clinicians must pay careful attention to pediatric TB, regardless of season, to prevent delayed diagnosis.

We found that the epidemic monthly peaks for age groups 15–24 and 25–44 years occurred from April to June and June to August, respectively (Fig. 3). These results are compatible with those of studies conducted in the United Kingdom [27], Spain [28, 36], India [25, 26], and Hong Kong indicating that seasonality for TB incidence is highest in late spring or early summer, owing to indoor crowding in the wintertime. This season-specific risk factor of TB incidence in these age groups might be similar to the conditions of TB cases in Japan. In addition, one of the main routes of TB detection in Japan is via regular health check-ups among schoolchildren and workers, which is regulated by the Industrial Safety and Health Act [37]. The fixed timing of annual health checkups in Japan may contribute to the seasonality of TB cases in these age groups. School-age children in Japan are required to receive a health checkup at the beginning of the school year, in April or May. In contrast, the timing of health checkups for adult workers is at the beginning of the fiscal year, which is usually in June for many Japanese companies. A regulated health checkup is provided, which includes a chest radiograph examination. New TB cases are commonly reported at the time of these regular checkups. Thus, the seasonal peaks of TB cases tend to match the timing of health checkups among school-age children and working populations. This result emphasizes the need to adopt measures for early diagnosis of TB among these active populations.

The incidence of TB among older adults in Japan is extremely high compared with other age groups (Table 1). A Japanese study indicated that the number of smear-positive pulmonary TB cases among patients aged ≥70 years increased by 2.5 times from 1980 to 2000, and most patients had been infected with TB bacilli in the past [38]. In the present study, epidemic peaks for TB cases among people aged 65–74 years tended to be longer than those for younger people and appeared from late spring to early winter. However, the monthly peak in male groups started in March and declined in September and November; monthly peaks in female groups started in June and did not decline until December. This finding may be explained by reactivation of latent TB infection in older adults, irrespective of sex, owing to aging, biological senescence, and immunologic change. In addition, a variety of viral and bacterial respiratory infectious diseases exhibit winter seasonality [39], which may increase susceptibility of individuals to infection by suppressing host defense capacity and concealing disease manifestation in patients with latent TB [8]. The reported findings of experimental studies imply that the number of natural killer cells and CD4 T-cells increase in winter and are associated with increased blood levels of interleukin-6 [40]. This could be a reason that the lowest epidemic peaks were observed in January and February in all age groups ≥15 years in our study (Fig. 3). In addition, seasonality causes a delay in health care-seeking behaviors, especially for male patients aged 65–74 years, who tend to prefer staying at home in the winter season. As a result, the timing of epidemic monthly peaks in male patients within this age group starts in March (e.g., the start of warmer weather in early spring), whereas those in females begins in June. Furthermore, a study suggested that spring surges in TB cases may be owing to delays in the diagnosis of wintertime disease that presents as common seasonal symptoms, including cough and fever with viral upper respiratory infections [7]. Therefore, it can be speculated that the proper diagnosis of TB during its course would be delayed more frequently in winter [22].

The limitations of the present study were owing to the nature of an ecological study that used the national surveillance data. We were unable to directly address the cause of seasonality in the incidence of TB. However, we evaluated nationwide data from the previous 9 years. Although TB seasonality has been previously reported in different counties and regions, our results are a unique report from Japan, an industrialized country with the most rapidly aging population in the world and the Intermediate TB burden.

## Conclusions

Pulmonary TB in Japan exhibits seasonality among people older than 15 years of age, with a peak in summer. Monthly trends differ according to age groups. The epidemic period tends to be longer with increasing age in patients who may have reactivated latent TB infection. Health checkups in young adults, reactivation of latent TB infection with aging, and lifestyle habits among older adults may be season-specific risk factors for TB incidence. The early detection of TB, and enhancing clinical practice in TB specialized and non-TB specialized hospitals would be crucial for improving the clinical management on TB patients in Japan. Our results will assist in further investigations to clarify the specific factors leading to seasonality in TB incidence.

## Abbreviation

TB: tuberculosis

## References

[1] World Health Organization. Global Tuberculosis Report 2015. http://apps.who.int/iris/bitstream/10665/250441/1/9789241565394-eng.pdf?ua=1. Accessed 1 August 2017.

[2] Ministry of Health, Labour and Welfare-Japan. Available at http://www.mhlw.go.jp/stf/seisakunitsuite/bunya/0000175095.html. Accessed 4 January 2017. (Japanese).

[3] Japan Anti-Tuberculosis Association. Statistics of Tuberculosis 2015. Available at http://www.jata.or.jp/rit/ekigaku/toukei/nenpou/http://www.jata.or.jp/rit/ekigaku/. Accessed April 1, 2017.

[4] Wubuli A, Li Y, Xue F, Yao X, Upur H, Wushouer Q (2017). Seasonality of active tuberculosis notification from 2005 to 2014 in Xinjiang, China. PLoS One.

[5] Yang X, Duan Q, Wang J, Zhang Z, Jiang G (2014). Seasonal variation of newly reported pulmonary tuberculosis cases from 2004 to 2013 in Wuhan, China. PLoS One.

[6] Koh GC, Hawthorne G, Turner AM, Kunst H, Dedicoat M (2013). Tuberculosis incidence correlates with sunshine: an ecological 28-year time series study. PLoS One.

[7] Maclachlan JH, Lavender CJ, Cowie BC (2012). Effect of latitude on seasonality of tuberculosis, Australia, 2002-2011. Emerg Infect Dis.

[8] Willis MD, Winston CA, Heilig CM, Cain KP, Walter ND, Mac Kenzie WR (2012). Seasonality of tuberculosis in the United States, 1993-2008. Clin Infect Dis.

[9] Gibney KB, MacGregor L, Leder K, Torresi J, Marshall C, Ebeling PR (2008). Vitamin D deficiency is associated with tuberculosis and latent tuberculosis infection in immigrants from sub-Saharan Africa. Clin Infect Dis.

[10] Talat N, Perry S, Parsonnet J, Dawood G, Hussain R (2010). Vitamin D deficiency and tuberculosis progression. Emerg Infect Dis.

[11] Martineau AR, Nhamoyebonde S, Oni T, Rangaka MX, Marais S, Bangani N (2011). Reciprocal seasonal variation in vitamin D status and tuberculosis reportcations in Cape Town, South Africa. Proc Natl Acad Sci U S A.

[12] Nagayama N, Ohmori M (2006). Seasonality in various forms of tuberculosis. Int J Tuberc Lung Dis.

[13] Reports for Tuberculosis registrant information. Ministry of Health, Labour and Welfare. Available at http://www.mhlw.go.jp/stf/seisakunitsuite/bunya/0000132952.html. Accessed 10 December 2016. (Japanese).

[14] The Tuberculosis Surveillance Center. Monthly reports. http://www.jata.or.jp/rit/ekigaku/. Accessed 10 December 2016. (Japanese).

[15] Ministry of Health, Labour and Welfare. Tuberculosis. Available at http://www.mhlw.go.jp/bunya/kenkou/kekkaku-kansenshou11/01-02-02.html. Accessed 27 December 2016. (Japanese).

[16] The Japanese Society for Tuberculosis. Guideline of Clinical Management on Tuberculosis (the 3^rd^ edition). Nankodo, Tokyo. 2015 (Japanese).

[17] Japan Meteorological Agency. Terminologies relating to time. Available at http://www.jma.go.jp/jma/indexe.html. Accessed 4 January 2017. (Japanese).

[18] Rogers JH (1977). A significance tests for cyclic trends in incidence data. Biometrika.

[19] Demirkok SS, Basaranoglu M, Coker E, Karayel T (2007). Seasonality of the onset of symptoms, tuberculin test anergy and Kveim positive reaction in a large cohort of patients with sarcoidosis. Respirology.

[20] Manabe T, Yamaoka K, Tango T, Binh NG, Co DX, Tuan ND (2016). Chronological, geographical, and seasonal trends of human cases of avian influenza a (H5N1) in Vietnam, 2003-2014: a spatial analysis. BMC Infect Dis.

[21] Naranbat N, Nymadawa P, Schopfer K, Rieder HL (2009). Seasonality of tuberculosis in an eastern-Asian country with an extreme continental climate. Eur Respir J.

[22] Akhtar S, Mohammad HG (2008). Seasonality in pulmonary tuberculosis among migrant workers entering Kuwait. BMC Infect Dis.

[23] Korthals Altes H, Kremer K, Erkens C, van Soolingen D, Wallinga J (2012). Tuberculosis seasonality in the Netherlands differs between natives and non-natives: a role for vitamin D deficiency?. Int J Tuberc Lung Dis..

[24] Parrinello CM, Crossa A, Harris TG (2012). Seasonality of tuberculosis in new York City, 1990-2007. Int J Tuberc Lung Dis.

[25] Behera D, Sharma PP (2011). A retrospective study of seasonal variation in the number of cases diagnosed at a tertiary care tuberculosis hospital. Indian J Chest Dis Allied Sci.

[26] Thorpe LE, Frieden TR, Laserson KF, Wells C, Khatri GR (2004). Seasonality of tuberculosis in India: is it real and what does it tell us?. Lancet..

[27] Douglas AS, Strachan DP, Maxwell JD (1996). Seasonality of tuberculosis: the reverse of other respiratory diseases in the UK. Thorax..

[28] Luquero FJ, Sanchez-Padilla E, Simon-Soria F, Eiros JM, Golub JE (2008). Trend and seasonality of tuberculosis in Spain, 1996-2004. Int J Tuberc Lung Dis.

[29] Leung CC, Yew WW, Chan TY, Tam CM, Chan CY, Chan CK (2005). Seasonal pattern of tuberculosis in Hong Kong. Int J Epidemiol.

[30] Guo C, Du Y, Shen SQ, Lao XQ, Qian J, Ou CQ (2017). Spatiotemporal analysis of tuberculosis incidence and its associated factors in mainland China. Epidemiol Infect.

[31] World Health Organization. World Health Statistics 2016: Monitoring Health for the SDGs. Available at http://www.who.int/gho/publications/world_health_statistics/2016/en/. Accessed January 5, 2018.

[32] Mori T (2002). 100 years of tuberculosis outbreak and measurements in Japan. Journal of Japan Society of Internal Medicine.

[33] Aoki M (1988). Some recent aspects of tuberculosis infection in Japan (1). Kekkaku.

[34] Mori T (2004). Reform of Japan's NTP and its technical perspectives. Kekkaku.

[35] Snène H, Berraies A, Hamdi B, Ammar J, Ouali H, Hamzaoui A (2016). Childhood tuberculosis: a descriptive study in a pneumo-pediatrics department in Tunisia. Tunis Med.

[36] Ríos M, García JM, Sánchez JA, Pérez D (2000). A statistical analysis of the seasonality in pulmonary tuberculosis. Eur J Epidemiol.

[37] Industrial Safety and Health Act (Act No. 57 of June 8, 1972). Available at http://www.japaneselawtranslation.go.jp/law/detail_main?re=02&ia=03&vm=02&id=1926

[38] Ohmori M, Ishikawa N, Yoshiyama T, Uchimura K, Aoki M, Mori T (2002). Current epidemiological trend of tuberculosis in Japan. Int J Tuberc Lung Dis.

[39] Li XX, Wang LX, Zhang H, Du X, Jiang SW, Shen T (2013). Seasonal variations in reportcation of active tuberculosis cases in China, 2005-2012. PLoS One.

[40] Maes M, Stevens W, Scharpé S, Bosmans E, De Meyer F, D’Hondt P (1994). Seasonal variation in peripheral blood leukocyte subsets and in serum interleukin-6, and soluble interleukin-2 and -6 receptor concentrations in normal volunteers. Experientia.

